# The Intestinal Microbiota Composition in Early and Late Stages of Diabetic Kidney Disease

**DOI:** 10.1128/spectrum.00382-23

**Published:** 2023-06-21

**Authors:** Li Zhang, Qi-Yu Lu, Hao Wu, Yan-Li Cheng, Jing Kang, Zhong-Gao Xu

**Affiliations:** a Department of Nephrology, The First Hospital of Jilin University, Changchun, Jilin Province, China; b Department of Thyroid Surgery, General surgery center, The First Hospital of Jilin University, Changchun, Jilin Province, China; c Department of Endocrinology, The Second Hospital of Jilin University, Changchun, Jilin Province, China; Universita degli Studi di Parma

**Keywords:** diabetic kidney disease, gut microbiome, dysbiosis, biomarkers

## Abstract

Many studies have suggested that gut microbiota dysbiosis may be one of the pathogenesis factors of diabetes mellitus (DM), while it is not clear whether it is involved in the development of diabetic kidney diseases (DKD). The objective of this study was to determine bacterial taxa biomarkers during the progression of DKD by investigating bacterial compositional changes in early and late DKD. 16S rRNA gene sequencing was performed on fecal samples, including the diabetes mellitus (DM), DNa (early DKD), and DNb (late DKD) groups. Taxonomic annotation of microbial composition was performed. Samples were sequenced on the Illumina NovaSeq platform. At the genus level, we found counts of *Fusobacterium*, *Parabacteroides*, and *Ruminococcus_gnavus* were significantly elevated both in the DNa group (*P = *0.0001, 0.0007, and 0.0174, respectively) and the DNb group (*P < *0.0001, 0.0012, and 0.0003, respectively) compared with those in the DM group. Only the level of *Agathobacter* was significantly decreased in the DNa group than the DM group and in the DNb group than the DNa group. Counts of *Prevotella_9*, *Roseburia* were significantly decreased in the DNa group compared with those in the DM group (*P = *0.001 and 0.006, respectively) and in the DNb group compared with those in the DM group (*P < *0.0001 and 0.003, respectively). Levels of *Agathobacter, Prevotella_9, Lachnospira*, and *Roseburia* were positively correlated with an estimated glomerular filtration rate (eGFR), but negatively correlated with microalbuminuria (MAU), 24 h urinary protein quantity (24hUP), and serum creatinine (Scr). Moreover, the areas under the curve (AUCs) of *Agathobacter* and *Fusobacteria* were 83.33% and 80.77%, respectively, for the DM and DNa cohorts, respectively. Notably, the largest AUC for DNa and DNb cohorts was also that of *Agathobacter* at 83.60%. Gut microbiota dysbiosis was found in the early and late stages of DKD, especially in the early stage. *Agathobacter* may be the most promising intestinal bacteria biomarker that can help distinguish different stages of DKD.

**IMPORTANCE** It is not clear as to whether gut microbiota dysbiosis is involved in the progression of DKD. This study may be the first to explore gut microbiota compositional changes in diabetes, early-DKD, and late DKD. We identify different gut microbial characteristics during different stages of DKD. Gut microbiota dysbiosis is found in the early and late stages of DKD. *Agathobacter* may be the most promising intestinal bacteria biomarker that can help distinguish different stages of DKD, although further studies are warranted to illustrate these mechanisms.

## INTRODUCTION

Nowadays, the leading cause of end-stage renal disease (ESRD) is diabetic kidney disease (DKD) (1), which occurs in 30 to 40% of diabetes mellitus (DM) patients and whose global prevalence is increasing at an alarming rate (2, 3). Although hyperglycemia and hypertension are known to drive the onset and progression of DKD, strict glycemic control could not stop the progression of DKD to ESRD or death (4, 5). As the underlying mechanism of DKD pathogenesis has not been elucidated, there are no effective methods to prevent renal progression of DKD (6). Therefore, the elucidation of the mechanism and biomarkers of progression of DKD is an important unmet medical need.

Over the past decades, gut microbiota dysbiosis and the potential mechanisms involved in several diseases-diabetes, chronic kidney disease (CKD), inflammatory bowel disease, dyslipidemia, obesity, and cardiovascular disease-have become areas of intense research interest (7). In 2010, Larsen et al. first demonstrated that gut microbiota dysbiosis was linked to the severity of diabetes (8). Some studies reported intestinal dysbiosis existed in subjects with type 2 diabetes mellitus (T2DM) and prediabetic individuals (9, 10). To date, although many studies have investigated the role of intestinal microbiota in DM, few have explored the mechanisms involved in the progression of DKD. Yu et al. had shown the differences in composition of the gut microbiome between patients with DKD and those with membranous nephropathy (11). Tao et al. compared microbiota compositions between the patients with the early stages of DKD and the patients with DM, and found differences in *Prevotella_9* and Escherichia*-Shigella* (12). In contrast, Lecamwasam et al. did not identify any obvious microbial differences between DM-associated early and late stages of CKD (13). Hence, it is not clear as to whether gut microbiota dysbiosis is involved in promoting the progression of DKD.

At present, there are many studies on the difference of gut microbiota between DM and normal control groups, which suggest that patients with DM show evidence of gut dysbiosis (8–10), while our study focuses on the role of intestinal microbes in the development from diabetes to diabetic nephropathy (DN). Therefore, the purpose of this study was to determine bacterial biomarkers and explore microbial involvement in the mechanism underlying the progression of DKD by comparing gut microbiota composition among DM, and early- and late-stage DKD subjects.

## RESULTS

### Study cohort.

We enrolled 88 participants and assigned them to a DM group (*n* = 30), DN group (*n* = 58)-divided into DNa group (*n* = 26), and DNb group (*n* = 32) according to estimated glomerular filtration rate (eGFR), from September, 2019 to November, 2021.

The baseline characteristics of the participants are summarized in Table 1. There were no obvious differences between the groups regarding aspects of age, gender, course of T2DM, body mass index, glycosylated hemoglobin (Hb1AC), triacylglycerol, or high density lipoprotein (HDL) between DM and DN-a or between DN-a and DN-b groups.

**TABLE 1:** Baseline characteristics of study individuals^*a*^

Variables	DN-a (*n* = 26)	DN-b (*n* = 32)	DM (*n* = 30)	P(DN-a versus DM)	P (DN-a versus DN-b)
Age, yrs	51.9 ± 8.3	57.6 ± 6.9	51.5 ± 5.7	0.919	0.051
Gender, Male (*n* %)	17 (65.4%)	23 (70.6%)	16 (53.3%)	0.422	0.776
BMI^*b*^, kg/m^2^	27.48 ± 4.90	26.10 ± 3.20	25.42 ± 3.09	0.131	0.364
Course of T2DM, yrs	8.35 ± 7.04	13.52 ± 8.03	7.9 ± 6.31	0.824	0.007
HbA1c^*c*^, %	7.21 ± 1.16	6.73 ± 1.16	8.42 ± 1.86	0.011	0.097
TC^*d*^, mmol/L	6.09 ± 2.03	5.36 ± 1.47	5.11 ± 1.46	0.053	0.116
TG^*e*^, mmol/L	3.20 ± 2.92	1.78 ± 0.084	2.04 ± 1.62	0.047	0.03
HDL^*f*^, mmol/L	1.39 ± 0.60	1.25 ± 0.33	1.17 ± 0.39	0.075	0.506
LDL^*g*^, mmol/L	3.59 ± 1.28	3.14 ± 1.04	2.77 ± 1.12	0.018	0.162
SBP^*h*^, mmHg	155.1 ± 23.6	155.8 ± 20.3	124.9 ± 12.8	<0.0001	0.988
DBP^*i*^, mmHg	85.2 ± 10.8	84.1 ± 10.2	79.3 ± 7.9	0.027	0.506
sCr^*j*^, umol/L	95.70 ± 26.51	362.31 ± 221.44	70.77 ± 15.49	<0.0001	<0.0001
eGFR^*k*^, mL/min/1.73 m^2^	76.05 ± 22.23	20.40 ± 12.12	96.98 ± 16.27	<0.0001	<0.0001
Proteinuria, g/24 h	5.92 ± 5.30	7.55 ± 3.13	/	/	0.033

a Continuous data presented as mean ± standard deviation; categorical variables were presented as number (%). Independent-samples Mann-Whitney U test was used to evaluate continuous variables and Chi-square test was used to compare categorical variables between two groups. All the analyses were conducted with the SPSS program (version 18.0, Chicago, IL, USA), *P* < 0.05 was considered to be statistically significant.

b BMI, body mass index.

c HbA1c, Glycosylated Hemoglobin.

d TC, total cholesterol.

e TG, triacylglycerol.

f HDL, High Density Lipoprotein.

g LDL, Low Density Lipoprotein.

h SBP, Systolic blood pressure.

i DBP, Diastolic blood pressure.

j sCr, serum creatinine.

k eGFR, estimated glomerular filtration rate.

### Abundance feature analysis.

**FIG 1:**
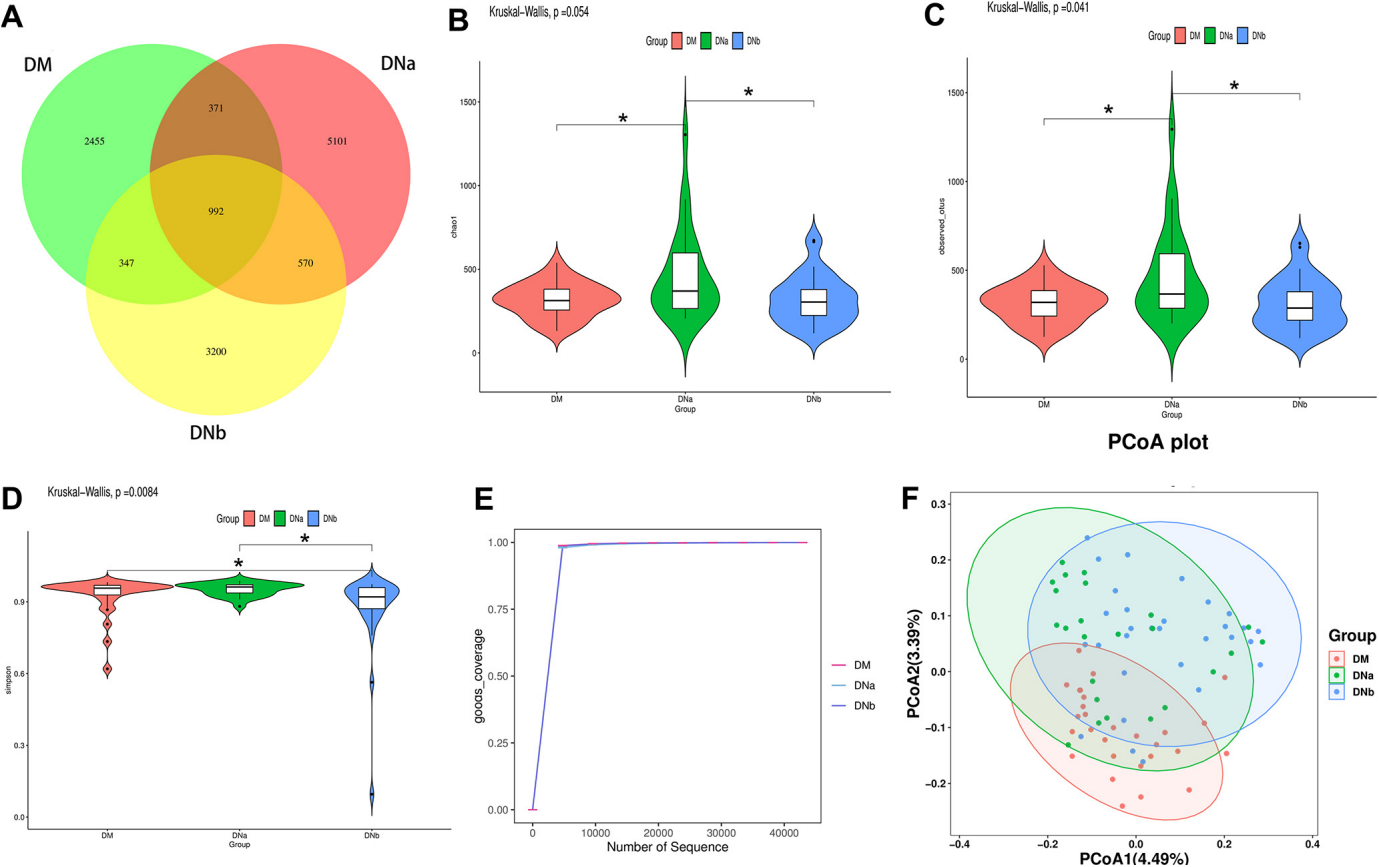
(A) Venn diagram displaying the degree of overlap of bacterial OTUs among DM, DNa, and DNb groups. (B) The violin plot of Chao1. (C) The violin plot of observed outs. (D) The violin plot of simpson. (E) The rarefaction curves of goods_coverage. (F) Principal Co-ordinates Analysis (PCoA) plot of the unweighted UniFrac distances revealed clustering of patients in 3 groups.

We analyzed all the DNA samples using 16S rRNA gene amplification and sequenced the products. Using the eigenvalue abundance table, the number of common features was calculated for each group, and the numbers of common and unique features of each group were visually presented using Venn diagrams. To show the differences more clearly between 3 experimental groups, we showed the Venn diagrams of the 3 groups (Fig. 1A).

We used alpha and beta diversity to explore biological heterogeneity and total diversity. Alpha diversity is mostly expressed by the values of Chao1, observed species, Good’s coverage, and Simpson indices. The violin plots of Chao1, observed species, and Simpson indices are shown in Fig. 1B, C, and D, respectively. Thus, there were significant differences in alpha diversity of the bacterial community between the 3 groups. The rarefaction curves of Goods coverage is shown in Fig. 1E. Except for several samples of the DNb group, our samples showed considerable richness and evenness in their microbial contents. The result of principal coordinate analysis (PCoA) is shown in Fig. 1F, indicating some different species discrepancy from environmental communities.

### Alterations in the fecal microbia composition associated with DKD.

**FIG 2:**
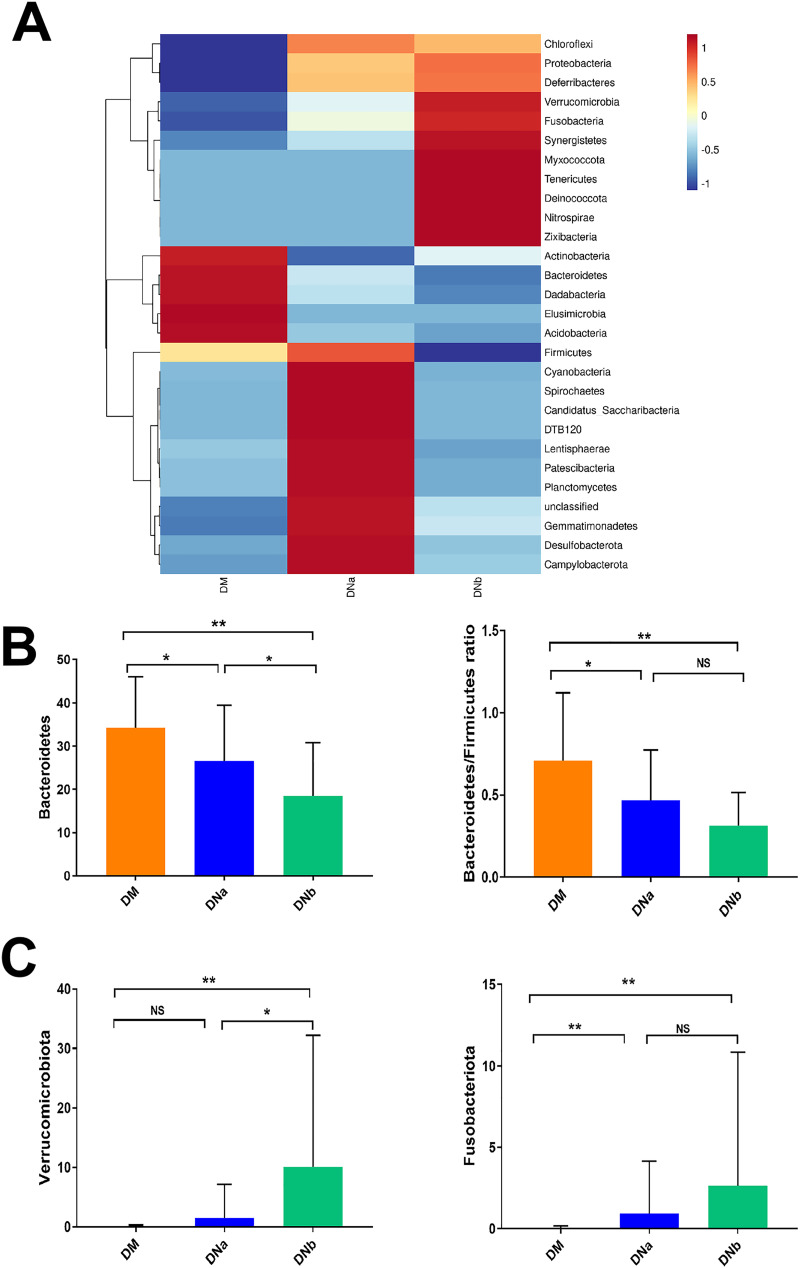
(A) Heatmap of relative abundance composition among DM, DNa, and DNb groups at the phylum level. (B)The bar graphs of relative abundance comparison of *Bacteroidetes* and *Bacteroidetes*/*Firmicutes* ratio among 3 groups. (C) The bar graphs of relative abundance comparison of *Verrucomicrobia* and *Fusobacteria* Pylum among 3 groups. NS means not significant, *, *P < *0.05 and **, *P < *0.001.

Heatmaps of fecal microbiota composition at the phylum level in the 3 groups are presented in Fig. 2A. Fecal microbia phyla composition between the DM, DNa, and DNb cohorts were quite different such as *Bacteroidete*s, *Firmicutes*, *Fusobacteriota*, and *Actinobacteriota*. Phylum *Bacteroidetes* was significantly decreased in the DNa and DNb groups, respectively, compared with the DM group and DNa group. *Bacteroidetes*/*Firmicutes* ratio was also significantly lower in the DNa group than in the DM group, but not in the DNb group compared with the DNa group (Fig. 2B). Moreover, we found a significantly higher abundance of the phylum *Fusobacteria* both in the DNa and the DNb groups compared with the DM group. However, there was no difference between the DNa and DNb groups. Phylum *Verrucomicrobia* was significantly elevated in the DNb group compared to both the DM group and DNa group (Fig. 2C).

**FIG 3:**
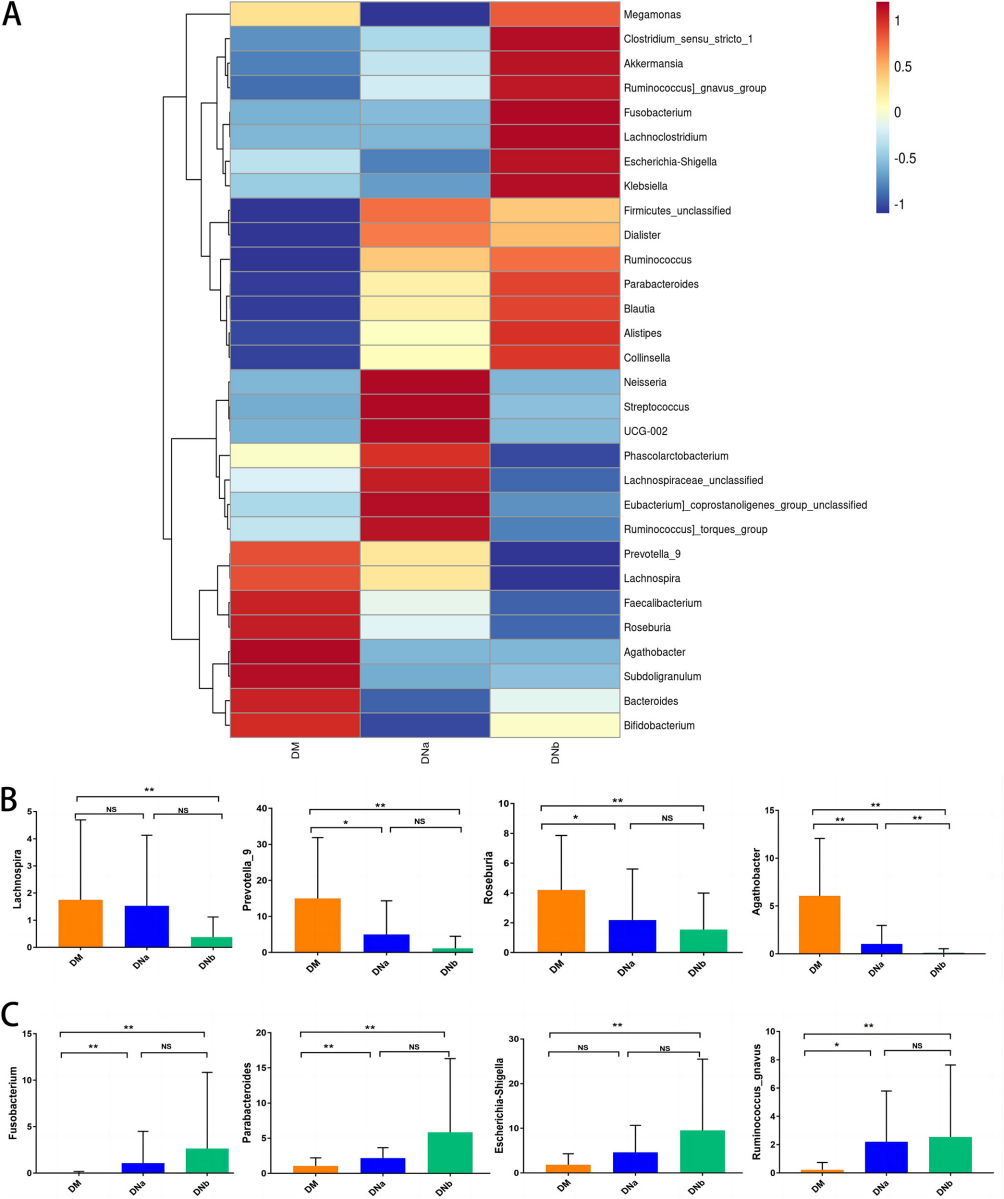
(A) Heatmap of relative abundance among DM, DNa, and DNb groups at the genus level. (B) The bar graphs of relative abundance comparison of abundance decreased genus in DNa or DNb groups compared with the DM group. (C) The bar graphs of relative abundance comparison of abundance elevated genus in DNa or DNb groups compared with DM group. NS means not significant, *, *P < *0.05 and **, *P < *0.001.

Heatmaps of fecal microbiota composition at the genus level in the DM, DNa, and DNb groups are presented in Fig. 3A. The composition of the high-abundance genera in the fecal microbiota was considerably different for all 3 groups. Only the level of *Agathobacter* was significantly decreased both in the DNa group than the DM group and in the DNb group than the DNa group. According to the bar graphs in Fig. 3B, counts of *Prevotella_9*, *Roseburia* were significantly decreased in the DNa group compared with those in the DM group (*P = *0.001 and 0.006, respectively) and in the DNb group compared with those in the DM group (*P* < 0.0001 and 0.003, respectively). Furthermore, the levels of gena *Fusobacterium*, *Parabacteroides*, and *Ruminococcus_gnavus* were significantly elevated in the DNa group (*P = *0.0001, 0.0007, and 0.0174, respectively) and the DNb group (*P < *0.0001, 0.0012, and 0.0003, respectively) compared with those in the DM group (Fig. 3C). We also summarized the alterations of gut microbiota composition in patients with DKD compared with DM (Table 2).

**TABLE 2:** Summary of gut microbiota alternation in patients with DKD

Phylum	Genus	DNa versus DM	DNb versus DM	DNb versus DNa
*Bacteroidetes*	*Parabacteroides*	**↑**	**↑**	NS
	*Prevotella_9*	**↓**	**↓**	NS
*Firmicutes*	*Agathobacter*	**↓**	**↓**	↓
	*Faecalibacterium*	↓	↓	NS
	*Lachnospira*	NS^*a*^	↓	NS
	*Roseburia*	↓	↓	NS
	*Ruminococcus_gnavus*	↑	↑	NS
*Fusobacteriota*	*Fusobacterium*	↑	↑	NS
*Proteobacteria*	Escherichia *-Shigella*	NS	↑	NS
*Bacteroidetes*/*Firmicutes*		↓	↓	NS

a NS means not significant.

**FIG 4:**
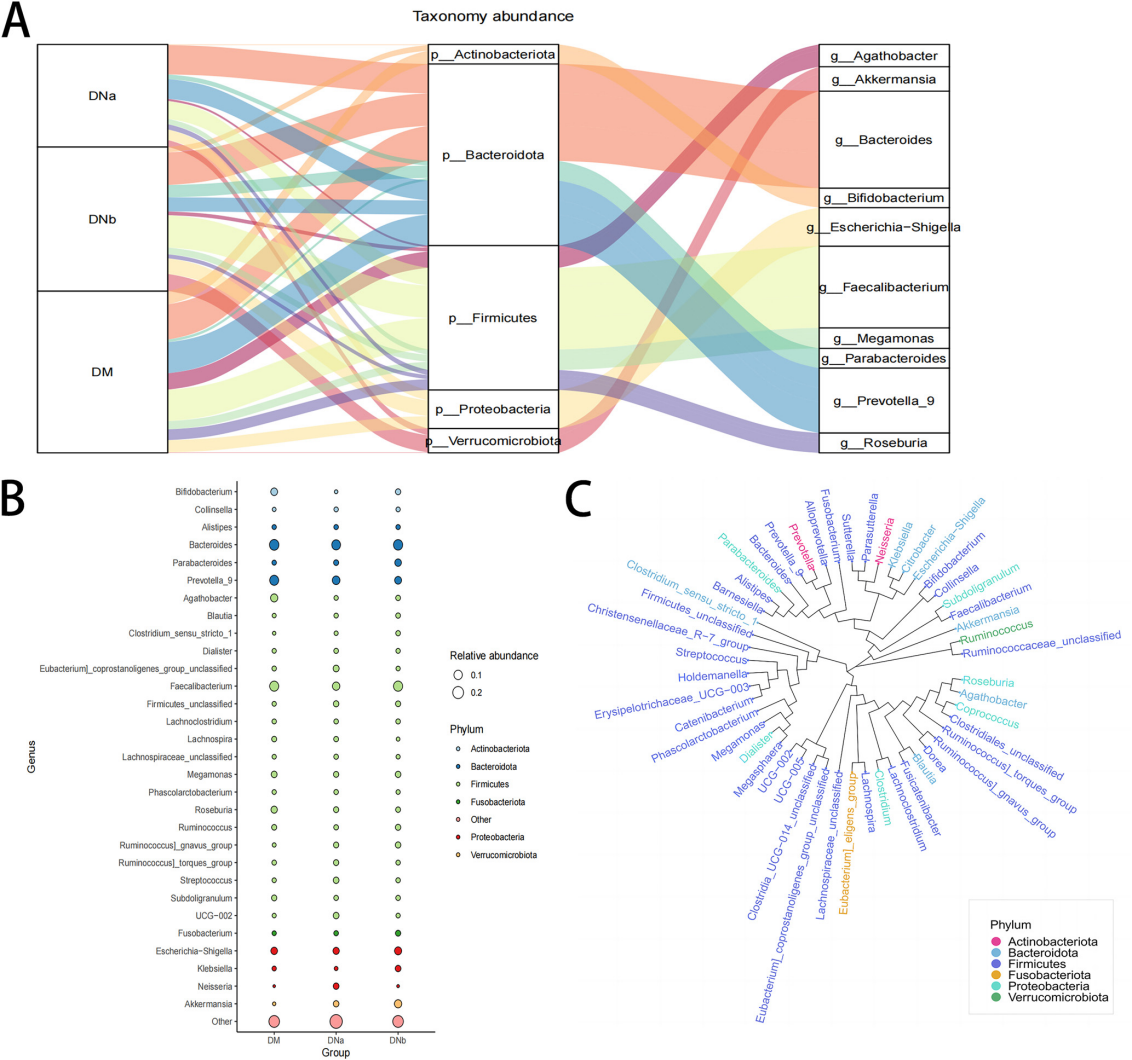
(A) Sankey plot shows the relative abundance of microbiota at the phylum level (middle) and genus level (right) corresponding to different groups (left). (B) Bubble plot shows species information and relative abundance (circle size) at the genus level for 3 groups, as well as the species information (circle color) for the corresponding phylum. (C) Phylotree of different gena.

To clearly demonstrate the differences at the genus and phylum levels, Sankey plots and bubble plots were graphed (Fig. 4A and 4B). These plots also showed a decrease in *Agathobacter* and *Prevotella_*9 levels in the DNb group. Phylotree showed *Agathobacter* and *Roseburia* might be evolutionarily closely related (Fig. 4C).

### Clinical values of gut microbiota markers.

**FIG 5:**
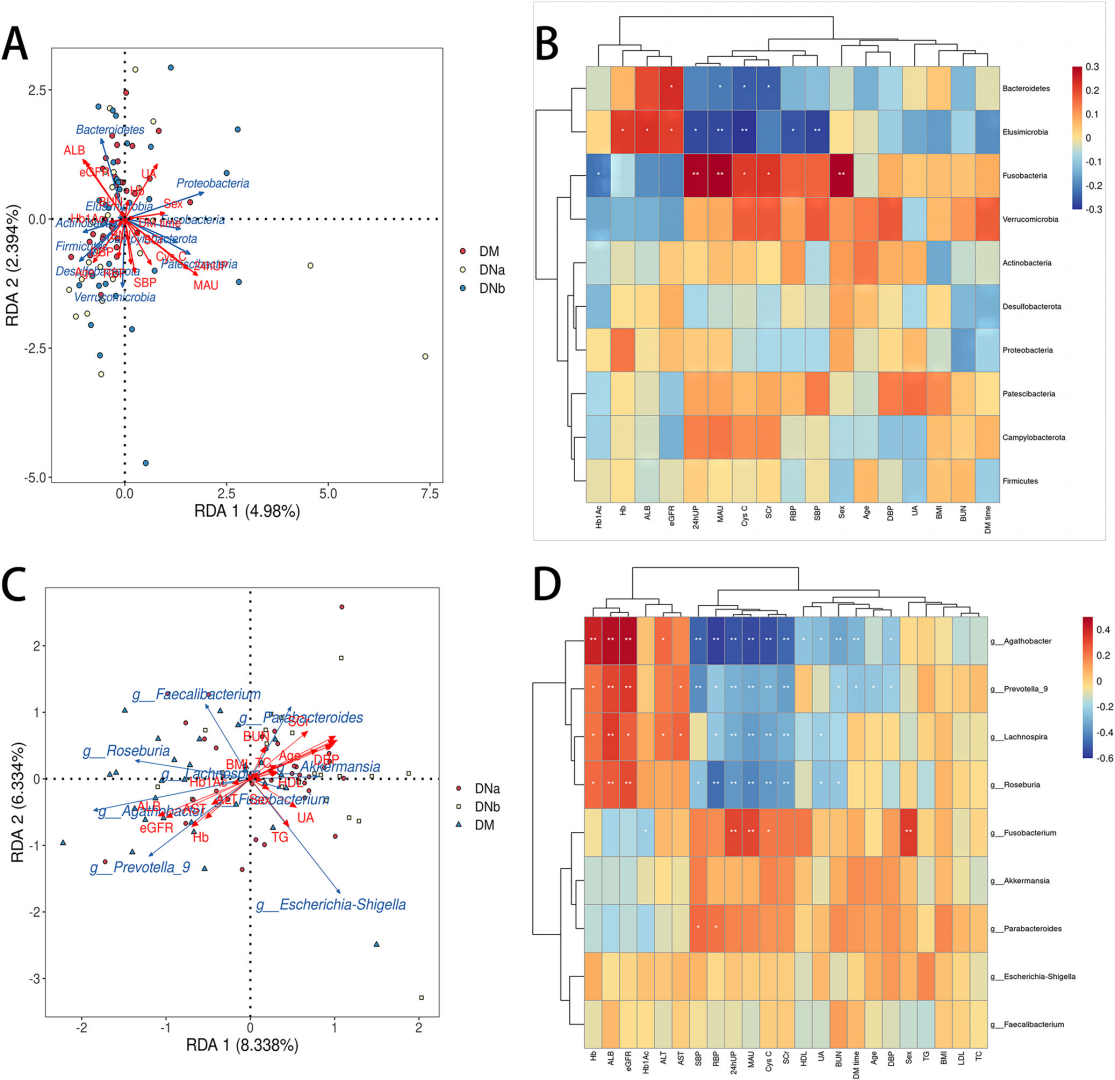
(A) RDA plot of bacterial diversity and clinical variables at the phylum level. (B) Correlation heatmap between the gut microbial biomarkers and clinical parameters at the phylum level. (C) RDA plot of bacterial diversity and clinical variables at the genus level. (D) Correlation heatmap between the gut microbial biomarkers and clinical parameters at the genus level.

The redundancy analysis (RDA) plot of bacterial diversity and clinical variables at the phylum level is shown in Fig. 5A. The correlation heatmap between the gut microbial biomarkers and clinical parameters at the phylum level is shown in Fig. 5B. The level of *Fusobacteria* was positively correlated with levels of microalbuminuria (MAU), 24 h urinary protein quantity (24hUP), serum cystatin C (CysC), and serum creatinine (Scr), but negatively correlated with Hb1AC. However, levels of *Bacteroidetes* were negatively correlated with MAU, Cys C, Scr, but positively correlated with eGFR.

The RDA plot and correlation heatmap at the genus level are presented in Fig. 5C and D. Levels of *Agathobacter*, *Prevotella_9*, *Lachnospira*, and *Roseburia* were positively correlated with haemoglobin (Hb), serum albumin (ALB), and eGFR, but negatively correlated with MAU, 24hUP, retinol binding protein (RBP), CysC, and Scr. The level of *Fusobacterium* was positively correlated with levels of MAU and 24hUP, but negatively correlated with Hb1AC. The level of *Ruminococcus_gnavus* group was positively correlated with levels of 24hUP, MAU, Cys C, Scr, and systolic and diastolic blood pressure. Moreover, *Fusobacterium* counts were significantly increased in male patients compared to female patients.

**FIG 6:**
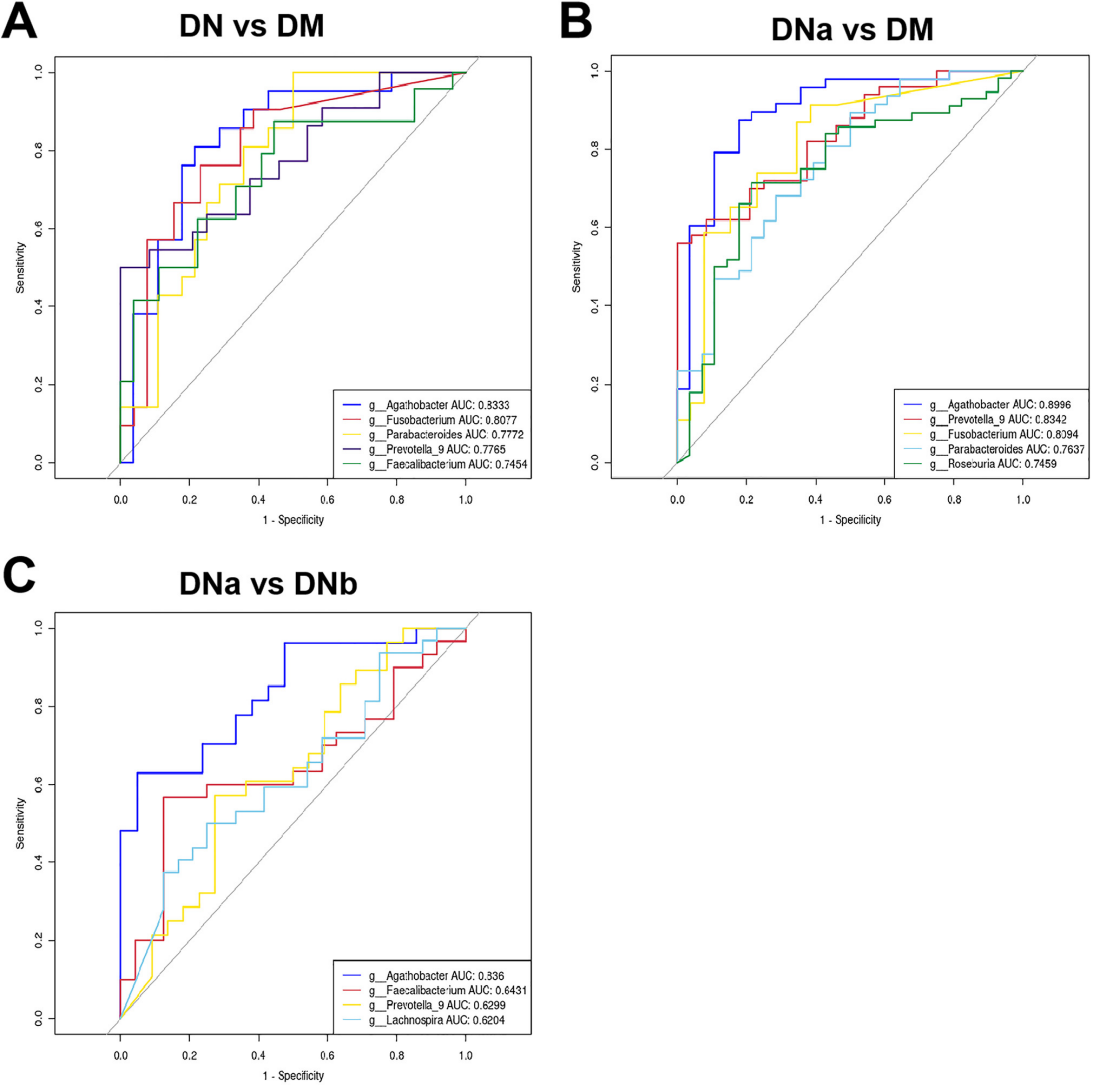
(A) ROC curve differentiating the DN cohort from the DM cohort based on the genus with excellent effect. (B) ROC curve differentiating the DNa cohort from the DM cohort based on the genus with excellent effect. (C) ROC curve classifying the DNb cohort from the DNa cohort based on the genus with excellent effect.

The receiver-operating characteristic (ROC) of the different cohorts are shown in Fig. 6A, B, and 6C. The areas under the curve (AUCs) of *Agathobacter* and *Prevotella_9* were 89.96% and 83.42%, respectively, for the DM and DN cohorts, respectively (DN group includes both DNa and DNb groups). The AUCs of *Agathobacter* and *Fusobacteria* were 83.33% and 80.77%, respectively, for the DM and DNa cohorts, respectively. Notably, the largest AUC for DNa and DNb cohorts was also that of *Agathobacter* at 83.60%.

## DISCUSSION

In the present study, we identified different gut microbial characteristics during different stages of DKD. Our study may be the first to explore the roles of gut microbial factors in DM, early DKD, and late DKD. Lecamwasam et al. (13) compared early and late diabetic CKD, whereas Tao et al. did not include DKD individuals in the late stage of CKD (12). To avoid the confounding effects of long-term dialysis, we did not include patients who had started regular hemodialysis or peritoneal dialysis. Moreover, we consistently observed a higher number of specific Amplicon Sequence Variants (ASVs) than that of common ASVs in all 3 groups. The number of ASVs in our study was much larger than that reported by Tao et al. (12). This could be attributed to our use of Quantitative Insights into Microbial Ecology 2 (QIIME2) instead of QIIME. In QIIME2, Divisive Amplicon Denoising Algorithm (DADA2) corrects the sequencing errors of amplicons through methods such as filtering, dereplication, and chimeric filtering to determine a higher number of true sequence variations instead of clustering the data based on sequence similarity. The feature table and feature sequence obtained by removing the background noise not only greatly improve the data accuracy and species resolution, but also ensure the reliability of the results (14).

We observed that phylum *Fusobacteria* levels were significantly elevated in the DNa and DNb groups compared with those of the DM group. However, there was no obvious difference in their levels between the DNa and DNb groups. These results are consistent with those reported by Lecamwasam et al. (13), who examined microbial composition and diversity in the early and late stages of diabetic CKD. They found that gut microbiota composition remains stable in individuals with both early- and late-stage diabetic CKD. Furthermore, in this study, we found that the level of phylum *Verrucomicrobia* was significantly elevated in the DNb group compared with the DM group. Khorraminezhad et al. reported that a high dairy intake decreased the abundance of *Verrucomicrobia* and had attributed this change to the glucose tolerance status (15). In agreement with our results, Salguero et al. (16) found that the relative abundance of *Verrucomicrobia* and *Fusobacteria* was significantly increased in patients with T2DM with CKD compared with healthy controls (16). As *Verrucomicrobia* and *Fusobacteria* are both Gram-negative bacteria, the increased lipopolysaccharide (LPS) levels may contribute to the progression of DKD (17–19), as LPS may induce inflammation by accelerating the activation of macrophages/monocytes and neutrophils (12). Nevertheless, Liu et al. demonstrated that *Verrucomicrobia* levels were negatively associated with the risks of DM (20).

Furthermore, we found the phylum *Bacteroidetes* and *Bacteroidetes*-to-*Firmicutes* ratio was significantly decreased in the DNa and DNb groups compared to the DM group. *Bacteroidetes* and *Firmicutes* were two main phyla in the human intestine (19). *Bacteroidetes* and *Firmicutes* were both decreased in CKD patients in Ren Z’s study (19). The abundances of most genera belonging to *Firmicutes*, including *Agathobacter*, *Faecalibacterium*, *Lachnospira* and *Roseburia*, were demonstrated to be significantly reduced in the DNa and the DNb groups compared with those in the DM group in the present study. Nevertheless, except for *Agathobacter*, other bacteria abundance was not significantly different between the early and late DKD groups in our study. Similarly, Xu et al. did not discover any significant differences in microbial composition between the low- and high-GFR groups of CKD (21). Moreover, we also found *Prevotella_*9 levels were significantly lower in the DNa and DNb groups compared to the levels in the DM group, which was consistent with the results reported by Tao et al. (12). *Prevotella_*9 has been found to decrease inflammation by producing short-chain fatty acids (SCFAs) in acute kidney injury (22). SCFAs-acetic acid, propionic acid, and butyric acid-can produce energy and nutrition elements for intestinal epithelial cells (23), lessen the severity of inflammation (24), maintain important functions of intestinal barriers (25), and immune cell homeostasis (26). Vaziri et al. (27) also found that decreased abundance of *Prevotellaceae* family may be associated with CKD. In addition, *Firmicutes* was also producing butyrate bacteria (28). Nevertheless, we demonstrated that Escherichia*-Shigella* were significantly enriched in the DNb group compared to the DM group; however, not significantly elevated in the DNa group compared with the DM group. In contrast to the significant increase in levels of Escherichia*-Shigella* in the DN group with early stage CKD reported by Tao et al. (12), Escherichia*-Shigella* was one of the *Proteobacteria* that was also Gram-negative bacteria (9). Escherichia*-Shigella* can produce ethanol and increase intestinal epithelial leakage, which in turn affects fatty acid metabolism (29, 30).

Additionally, we were trying to explore some gut microbial biomarkers during the progression process from DM to DKD. Most differences between DNa, DNb, and DM groups were certified in early DKD group compared with DM group in our study. We found a significant increase in levels of only *Agathobacter* in late DKD compared with those in early DKD. Thus, gut microbiota dysbiosis may mainly occur in early DKD (13). In order to deeply explore these bacteria’s clinical meanings, we did the correlation analysis between gut microorganisms and clinical parameters and ROC curves. We found that levels of *Agathobacter*, *Prevotella_*9, *Lachnospira*, and *Roseburia* were negatively correlated with the levels of MAU, 24hUP, RBP, CysC, and Scr, but positively correlated with eGFR. Furthermore, *Agathobacter* levels were positively correlated with the levels of HDL, uric acid, and blood urea nitrogen (BUN), as well as the duration of DM and hypertension. According to the AUC values of the ROC curves in the present study, *Agathobacter* levels might be the one with the most meaningful parameters to distinguish DKD from DM, early DKD from DM, or even late DKD from early DKD; hence, *Agathobacter* may be the most promising microbial biomarker for DKD. Indeed, *Agathobacter* are anaerobic, Gram-positive bacteria and may have some links to the renal damage in Henoch–Schönlein purpura (31). *Agathobacter* levels also have been found to be increased in young depressive adults after the intake of flavonoid-rich orange juice. Thus, we hypothesize that gut microbiota dysbiosis could be altered by changing the diet as a novel complementary therapy for DKD. Therefore, we concluded that gut microbiota dysbiosis occurred mainly in the early stage of DKD. This highlights the importance of maintaining gut microbial balance by using probiotics, prebiotics, or changing dietary habits especially in the early stage of DKD. We did not know whether the gut microbiota compositional changes drives DKD because of multiple comorbidities such as obesity and hypertension, which can also be affected by diet (19, 32). It seems to be plausible that gut microbiota and kidney diseases may be influenced by each other (19, 33).

Although our study may be the first to explore gut microbiota compositional changes in diabetes, early-DKD, and late DKD, it still has several limitations. Firstly, the groups included in our study encompassed small sample sizes. We present only 3 group results. Another limitation is that the CKD groups are divided into only 2 groups, and we should divide them into more detailed groups. Lastly, this was a cross-sectional study cohort, and was not studied longitudinally. Thus, we should conduct a longitudinal study with a large sample size and have more detailed groups in the future.

In conclusion, gut microbiota dysbiosis was found in the early and late stages of DKD, especially in the early stage. *Agathobacter* may be the most promising intestinal bacteria biomarker that can help distinguish different stages of DKD. However, we need more studies with larger sample sizes to confirm this in the future.

## MATERIALS AND METHODS

### Ethics statement.

The studies involving human participants were reviewed and approved by the Ethics Committee of The First hospital of Jilin University. Written informed consent to participate in the study were provided by the patients.

### Participants.

This cross-sectional study enrolled 88 participants. Inclusion criteria were: (i) Age 30 to 69 years; no special dietary habits; (ii) DM group: A clinical history of at least 1 month since the diagnosis of T2DM; without any kidney disease history: negative albuminuria and normal kidney function. Albuminuria was evaluated by measuring the urinary albumin-to-creatinine ratio ≥ 30 mg/g (34); (iii) DKD groups: DKD or DN was defined as T2DM with the presence of albuminuria, impaired glomerular filtration rate (GFR), or both (35). The CKD stages 1 to 5 were defined by Alan Go et al. (36). Therefore, we regarded CKD stages 1 to 3a as early stage CKD (DNa group), and CKD stages 3b to 5 as late-stage CKD (DNb group) (13). Exclusion criteria: (i) the use of any antibiotics within 1 month of enrollment or of probiotics or prebiotics within 1 week of enrollment; (ii) hematochezia, severe diarrhea, and other gastrointestinal diseases; (iii) known systemic diseases that may affect intestinal microbiota composition such as tumors and liver cirrhosis; (iv) patients in stage 5 CKD who started hemodialysis lasting more than 1 week.

The 88 participants included in the study were categorized into 3 groups: DM group (*n* = 30), DNa group (*n* = 26), and DNb group (*n* = 32). All the subjects agreed to provide a stool sample and undergo routine blood and urine tests during hospitalization or outpatient visits. Data collection included information on medical comorbidities, blood pressure, duration of diabetes, and medicines. The study was approved by the Human Research Ethics Committee of The First Hospital of Jilin University. All methods were performed in accordance with the relevant guidelines and regulations.

### Stool collection.

All the stool samples were acquired and stored using a Stool Storage Kit (Cat. No.:LS-R-P-007, Longsee, China), which contains a fluid to protect genomic DNA. All the samples were stored at −80°C until DNA extraction.

### PCR amplification and 16S rDNA sequencing.

DNA extraction was conducted according to the instructions of the E.Z.N.A. Stool DNA Kit (D4015, Omega, Inc.). We performed PCR and used products from LC-Bio Technology Co., Ltd. The bacterial 16S rRNA genes of each DNA sample were amplified by using a primer set specific for the V3-V4 variable region of the 16S rRNA gene with the general primers 805R (5′-GACTACHVGGGTATCTAATCC-3′) and 341F (5′-CCTACGGGNGGCWG CAG-3′) (37). PCR was conducted according to the protocol. We used Qubit (Invitrogen) to quantify the PCR products. The Agilent 2100 Bioanalyzer (Agilent) and the Library Quantification Kit for Illumina (Kapa Biosciences, Woburn) were respectively applied for assessing the size and number of the amplicon library. We sequenced the libraries on the NovaSeq PE 250 platform.

### Sequencing data analysis.

We sequenced the samples on the Illumina NovaSeq platform provided by LC-Bio according to the instructions. Quality-filtering was conducted on the raw reads to get clean tags of high-quality by fqtrim (v0.94). Vsearch software (v2.3.4) was used for filtering the chimeric sequences. The feature table and feature sequences were acquired after dereplication through DADA2. The core of DADA2 is denoising. The ASVs table was constructed through the concept of amplicon sequence variants (38, 39). Alpha diversity and beta diversity analyses were conducted to assess the discrepancy in bacterial diversity between the studied groups. The complexity of species diversity was analyzed through alpha diversity with 5 indices: observed species, Chao1, Good’s coverage, Shannon, and Simpson. All these indices, as well as beta diversity, were calculated for our samples using QIIME2 (40). Beta diversity analysis usually begins by calculating the distance matrix between environmental samples. The differences between samples were analyzed by PCOA. The graphs were plotted using R package (v3.5.2).

### Stastical analyses.

Differences of clinical characteristics between the 3 groups were judged by Fisher's exact test or Pearson’s Chi-square test. We performed Pearson’s rank correlation analysis to evaluate the correlations between bacteria abundance or between bacteria abundance and clinical characteristics. *P < *0.05 for all statistical tests was considered to be significant. Non-parametric Wilcoxon test was used for constructing heatmaps at the genus/phylum level. ROC analysis was performed using the OmicStudio tools. Other diagrams were drawn using the R version 3.5.2 (Vienna, Austria) or Graph Pad Prism 7 software.

### Data availability.

All data associated with this study are available in the main text. The names of the repository/repositories and accession number(s) can be found below: https://www.ncbi.nlm.nih.gov/bioproject/PRJNA824185.
